# Supplementary material for: Continental-scale associations of Arabidopsis thaliana phyllosphere members with host genotype and drought
Source: Nat Microbiol. 2024 Sep 6;9(10):2748–58. doi: 10.1038/s41564-024-01773-z (PMC11457713; doi:10.1038/s41564-024-01773-z)
Supplement: Supplementary file 1 — Reporting Summary [file 41564_2024_1773_MOESM1_ESM.pdf]

nature research

Corresponding author(s): Detlef Weigel

Last updated by author(s): 5/11/2024

Reporting Summary

Nature Research wishes to improve the reproducibility of the work that we publish. This form provides structure for consistency and transparency in reporting. For further information on Nature Research policies, see our Editorial Policies and the Editorial Policy Checklist.

Statistics

For all statistical analyses, confirm that the following items are present in the figure legend, table legend, main text, or Methods section.

n/a	Confirmed	
☐	☒	The exact sample size (*n*) for each experimental group/condition, given as a discrete number and unit of measurement
☐	☒	A statement on whether measurements were taken from distinct samples or whether the same sample was measured repeatedly
☐	☒	The statistical test(s) used AND whether they are one- or two-sided<br>*Only common tests should be described solely by name; describe more complex techniques in the Methods section.*
☐	☒	A description of all covariates tested
☐	☒	A description of any assumptions or corrections, such as tests of normality and adjustment for multiple comparisons
☐	☒	A full description of the statistical parameters including central tendency (e.g. means) or other basic estimates (e.g. regression coefficient) AND variation (e.g. standard deviation) or associated estimates of uncertainty (e.g. confidence intervals)
☐	☒	For null hypothesis testing, the test statistic (e.g. F, t, r) with confidence intervals, effect sizes, degrees of freedom and P value noted<br>*Give P values as exact values whenever suitable.*
☒	☐	For Bayesian analysis, information on the choice of priors and Markov chain Monte Carlo settings
☒	☐	For hierarchical and complex designs, identification of the appropriate level for tests and full reporting of outcomes
☐	☒	Estimates of effect sizes (e.g. Cohen's d, Pearson's r), indicating how they were calculated

Our web collection on statistics for biologists contains articles on many of the points above.

Software and code

Policy information about availability of computer code

Data collection	usearch (version 11), DADA2 (version 1.10.1), DECIPHER (version 2.8.1), gemma (version 0.98.3), GATK (version 3. 5), SEEKIN (version 3.0), R packages used: fpc (version 2.2.9), cluster (version 2.1.2), MuMIn (version 1.43.1), EIGENSOFT (version 6), edgeR (version 3.28.1), caret47 (version 6.0-86), automap (version 1.0-14), fasttreeMP (version 2.1.11)
Data analysis	Scripts for data processing, analyses and figure generation can be accessed at https://github. com/tkarasov/pathodopsis.

For manuscripts utilizing custom algorithms or software that are central to the research but not yet described in published literature, software must be made available to editors and reviewers. We strongly encourage code deposition in a community repository (e.g. GitHub). See the Nature Research guidelines for submitting code & software for further information.

Data

Policy information about availability of data

All manuscripts must include a data availability statement. This statement should provide the following information, where applicable:

- Accession codes, unique identifiers, or web links for publicly available datasets
- A list of figures that have associated raw data
- A description of any restrictions on data availability

v3-v4 16S rDNA sequence data were deposited in the European Nucleotide Archive (ENA) under the Primary Accession ENA: PRJEB44379. Metadata and processed read data sets including phyloseq objects are available at Zenodo under DOI 10.5281/zenodo.5140512. The Silva database is available at https://www.arb-silva.de/documentation/release-132/.

Field-specific reporting

Please select the one below that is the best fit for your research. If you are not sure, read the appropriate sections before making your selection.

☐ Life sciences ☐ Behavioural & social sciences ☒ Ecological, evolutionary & environmental sciences

For a reference copy of the document with all sections, see nature.com/documents/nr-reporting-summary-flat.pdf

Ecological, evolutionary & environmental sciences study design

All studies must disclose on these points even when the disclosure is negative.

Study description	A. thaliana plants (and their associated microbiomes) were collected across Europe
Research sample	The research sample was the rosette of Arabidopsis thaliana (processed for host genotype and microbiome composition).
Sampling strategy	Previous work characterized the population structure and distribution of A. thaliana in Europe (Platt et al. 2010, TG Consortium, 2016). We sought
Data collection	Each collection team went to a specific region in Eurasia, and followed a systematic pipeline for sterile collection of plant tissue and processing.
Timing and spatial scale	Samples were collected in Winter-Spring 2018 across Europe
Data exclusions	Samples were excluded from analysis if we could not verify that the host of isolation was A. thaliana or Brassicaceae, or if the read output did not reach the specified standard of 1,000 16S rDNA reads per sample.
Reproducibility	Significant results were tested over several spatial scales, and confounding covariates were included in regression models (such as collection group or plate for processing).
Randomization	Samples were collected in regions by specific collection groups. While this confounds a perfectly random collection design, we conducted 17 collection trips and are able to include collection trip as a cofactor in models.
Blinding	Investigators had no prior knowledge of the distribution of microbes prior to this study.

Did the study involve field work? ☒ Yes ☐ No

Field work, collection and transport

Field conditions	These parameters differed across field sites (information provided in metadata supplementary material)
Location	We collected from several hundred locations. Information provided in metata files.
Access & import/export	Respective national authorities of all sampled countries Party to the Nagoya Protocol were contacted ahead of collections. Where needed, advised measures were taken and resulted in sampling and export permit KC3M-160/11. 04. 2018 (Bulgaria), ABSCH-IRCC-FR-253846-1 (France) and ABSCH-IRCC-ES-259169-1 (Spain).
Disturbance	Only the soil within 5cm of the plant was collected with the plant.

Reporting for specific materials, systems and methods

We require information from authors about some types of materials, experimental systems and methods used in many studies. Here, indicate whether each material, system or method listed is relevant to your study. If you are not sure if a list item applies to your research, read the appropriate section before selecting a response.

Materials & experimental systems

n/a	Involved in the study	
☒	☐	Antibodies
☒	☐	Eukaryotic cell lines
☒	☐	Palaeontology and archaeology
☒	☐	Animals and other organisms
☒	☐	Human research participants
☒	☐	Clinical data
☒	☐	Dual use research of concern

Methods

n/a	Involved in the study	
☒	☐	ChIP-seq
☒	☐	Flow cytometry
☒	☐	MRI-based neuroimaging
